# Numerical Simulations and the Design of Magnetic Field-Enhanced Electron Impact Ion Source with Hollow Cylinder Structure

**DOI:** 10.1155/2020/2809485

**Published:** 2020-01-25

**Authors:** Guochen Qi, Di Tian, Guolun Gao, Guangda Liu, Chunling Qiu, Tao Long

**Affiliations:** ^1^College of Instrumentation & Electrical Engineering, Jilin University, Changchun 130061, China; ^2^Beijing SHRIMP Center, Institute of Geology Chinese Academy of Geological Sciences, Beijing 100037, China

## Abstract

An electron impact ion source-adopted magnetic field-enhanced technology has been designed for enhancing the electron intensity and the ionization efficiency. Based on the ion optic focus mechanism, an electron impact ionization source was designed, and the electron entrance into the ionization chamber was designed with a hollow cylinder structure to improve the ion extraction efficiency. Numerical simulation and optimal geometry were optimized by SIMION 8.0 to provide higher electron intensity and ion transmission efficiency. To improve the electron intensity, the influence of the filament potential and magnetic intensity was investigated, and the values of 70 eV and 150 Gs were chosen in our apparatus. Based on the optimal parameters, the air in the lab and oxygen gas was detected by the homemade apparatus, and the ion intensity was detected in the positive and negative ion modes, respectively. The homemade electron impact ion source apparatus has the potential to enhance ionization efficiency applied in the mass spectrometer ionization source.

## 1. Introduction

Electron beam is a widely used source for chemical composition analysis [1] and material characterization instrument [2]. It has been applied in scanning electron microscopy (SEM) [3], transmission electron microscopy (TEM) [4], mass spectrometry (MS) [5], and so on [5, 6]. Among them, an electron impact (EI) ionization source, as electron spray ionization (ESI) and atmospheric pressure chemical ionization (APCI), is used as the ionization source in mass spectrometers [7–10], and the EI ionization source has become the standard ionization source in gas chromatography-mass spectrometry (GC-MS) [11]. An electron beam was released from the high-temperature cathode filament of the EI source, and then the electron beam was accelerated to ionize analyte molecules [12].

The released electron intensity from the cathode filament is an important indicator for evaluating the EI ionization source, which is related to the instrument performance [13]. The most direct way to improve the ionization efficiency is to increase the filament current of the EI ionization source. However, the lifetime of the filament will decrease rapidly. To increase electron intensity without reducing filament lifetime, two electron-emitting filaments were used [14]. In principle, this should at least double the number of ions emitted per atom in the vapor compared with a single electron-emitting filament.

One of the key components of instruments is the ionization source whose performance determines the sensitivity and resolution parameters. To enhance the electron intensity and analyte molecules' ionization efficiency, a magnet was placed behind the filament to create a homogeneous magnetic field inside the chamber. The electron trajectory was modified by the magnetic field as a result of the Lorentz force, thereby increasing the length of the electron trajectory through the ionization chamber. Previous studies have shown the critical role of magnetic field values in electron and ion trajectories for optimizing ionization [15–17].

The EI ionization source not only generates molecular ions but also introduces numerous ion dissociation processes due to the excess energy imparted to the neutrals. To reduce the fragment ions and generate a molecular ion, Ehrhardt and Straub used the electron beam with low electron energy and obtained the simple spectrum [18, 19]. But the effective collision cross section of electron and gas molecules was reduced as electron energy was reduced, which results in the decrease of analyte molecule ionization efficiency. To improve the ionization efficacy, Munson and Field first developed the chemical ionization (CI) source in 1966 [20]. Reagent gas molecules are ionized by electron ionization, which subsequently react with analyte molecules in the gas phase in order to achieve ionization, thus generating spectra exhibiting fewer fragment ion signals. However, the EI-CI was limited by the reaction of reagent and analyte molecules, where not all reactions can occur.

Under the high electron energy, the ion formation, ion dissociation, and fragmentation pathways are closely related to the structural information of molecules, and the structure of analytes can be elucidated with the specific rule. Moreover, the EI spectrum provides abundant fragment ion information, is highly reproducible, and is comparable across different instrumental platforms. Hence, the EI spectrum is ideal for database applications. The traditional way to ionize small molecules (up to ∼800 amu) is by colliding with energetic electrons (70 eV) in the highly diluted gas phase.

Recently, SIMION was used in the magnetic and electric field number simulation, and it has become possible to estimate the ionization efficiencies of ion sources and investigate new promising approaches to significantly improve ion production and extraction into the EI ionization source [21, 22].

The aim of this work is to find the optimal parameters of electrical and magnetic fields which correspond to the maximum. An EI ionization source equipped with a magnet has been designed for enhancing the electron intensity and the ionization efficiency. Based on the ion optic mechanism, we have designed the electron impact ion source, and the electron entrance into the ionization chamber was designed with a hollow cylinder structure to improve the electron transfer efficiency. Numerical simulation and optimal geometry were optimized by SIMION 8.0 to provide higher electron intensity and higher ion transmission efficiency. To improve the electron intensity, the influence of the filament potential and magnetic intensity was investigated. Based on optimal parameters, the air in the lab was detected by the homemade apparatus, and the ion intensity was detected in the positive and negative ion modes, respectively.

## 2. EI Ionization Source Simulation

**Figure 1:**
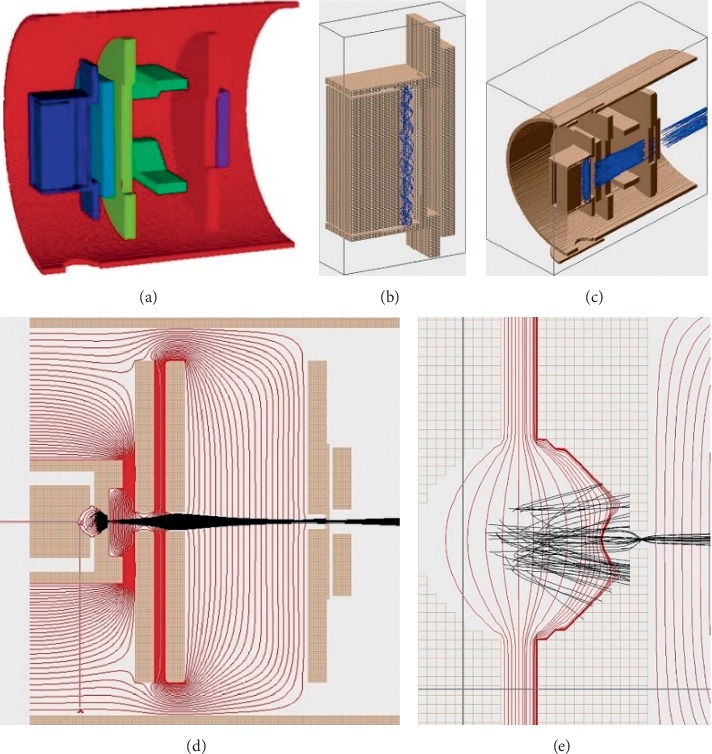
(a) Simulation structure of the designed EI ionization source. (b) Electron trajectory (blue lines) simulation of the designed EI ionization source. (c) Ion trajectory (blue lines) simulation of the designed EI ionization source. (d) Potential contours (red lines) and ion focus effect (black lines) in the EI ionization source. (e) Ion optic focus effect in the ionization chamber.

To investigate the designed EI ionization source, the electron movement and ion transmission were simulated by SIMION 8.0. The geometry of the designed EI ionization source is shown in Figure 1(a). Electron trajectories and ion trajectories were studied by computer simulations for better understanding and optimizing the processes of ion production and ion extraction as a function of the applied electrical and magnetic fields. Charged particles are characterized by helical trajectories due to the combined effects of the electrical and magnetic fields.

The system was simulated using 20 electrons (formed at the cathode filament position with diameter 1 mm circle distribution, fan-shaped emission) and 1000 ions (*m*/*z* 79) (formed in the cylinder ionization chamber with diameter 3 mm and height 23 mm). The initial ion energies were assumed to have a uniform distribution from 0 to 0.5 eV. The simulation parameter is shown in Supplementary Figure S1. The space charge has been shown to play a key role in the simulation of electron and ion trajectories. This effect is a result of the accumulation of charges due to the trapping of electrons, and it was taken into account using the POISSON module in SIMION 8.0. The effect of the magnetic field on the electron movement is shown in Figure 1(b), the effect of the focus electrode potential on the ion transmission is shown in Figure 1(c), potential contours (blue lines) for the ionization chamber are shown in Figure 1(d), and the ion optic focus effect in the ionization chamber is shown in Figure 1(e).

### 2.1. Ion Optic Lens Theory

**Figure 2:**
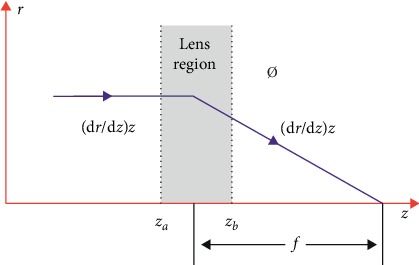
Diagram of the ion optic focus.

As for the electrostatic field (Figure 2), the electron or ion trajectory for the Gaussian movement can be written as

(1) $$\begin{array}{c} \sqrt{\emptyset }\frac{\text{d}}{\text{d}z}\left(\sqrt{\emptyset }\frac{\text{d}z}{\text{d}r}\right)=-\frac{\emptyset^{\prime \prime }}{4}r. \end{array}$$

Thus,

(2) $$\begin{array}{c} \text{d}\left(\sqrt{\emptyset }\frac{\text{d}r}{\text{d}z}\right)=-\frac{\emptyset^{\prime \prime }}{4\sqrt{\emptyset }}r\text{d}z. \end{array}$$

Applying integral calculus to equation (2), we get

(3) $$\begin{array}{c} \int_{z_{a}}^{z_{b}}\text{d}\left(\sqrt{\emptyset }\frac{\text{d}r}{\text{d}z}\right)=\int_{z_{a}}^{z_{b}}-\frac{\emptyset^{\prime \prime }}{4\sqrt{\emptyset }}r\text{d}z. \end{array}$$

Thus,

(4) $$\begin{array}{c} {\left.\sqrt{\emptyset }\frac{\text{d}r}{\text{d}z}\right|}_{z=z_{b}}-{\left.\sqrt{\emptyset }\frac{\text{d}r}{\text{d}z}\right|}_{z=z_{a}}=-\frac{1}{4}\int_{z_{a}}^{z_{b}}\frac{\emptyset^{\prime \prime }}{\sqrt{\emptyset }}r\text{d}z. \end{array}$$

Thus, the equation of the motion for the principal axial trajectory is

(5) $$\begin{array}{c} {\left.\frac{\text{d}r}{\text{d}z}\right|}_{z=z_{a}}=0,\\ {\left.\frac{\text{d}r}{\text{d}z}\right|}_{z=z_{b}}=r^{\prime }\left(z_{b}\right). \end{array}$$

In the electrostatic field region of the thin lens, *r* = *r* (*z*_*a*_) = *r*_*a*_ = constant, and the focal length can be calculated as

(6) $$\begin{array}{c} \frac{1}{f_{b}}=\frac{r^{\prime }\left(z_{b}\right)}{r_{a}}=\frac{1}{4\sqrt{\phi_{b}}}\int_{z_{a}}^{z_{b}}\frac{\phi^{\prime \prime }}{\sqrt{\phi }}dz. \end{array}$$

The objects' region is the isoelectric space, and ∅′(*z*_*a*_)=∅′(*z*_*b*_)=0.

The equation can be written as

(7) $$\begin{array}{c} \frac{1}{f_{b}}=\frac{1}{8\sqrt{\phi_{b}}}\int_{z_{a}}^{z_{b}}\frac{\phi^{\prime 2}}{\phi^{3/2}}\text{d}z. \end{array}$$

Likewise,

(8) $$\begin{array}{c} \frac{1}{f_{a}}=\frac{1}{8\sqrt{\phi_{a}}}\int_{z_{a}}^{z_{b}}\frac{\phi^{\prime 2}}{\phi^{3/2}}\text{d}z. \end{array}$$

Equations (7) and (8) are the theoretical base of the one-ion optic lens design. By adjusting the potential of the lens, the focal length of the object is adjusted, and the shape of the ion beam can be modulated.

## 3. Instrumentation

This setup is equipped with an EI ionization source implemented using a hot cathode filament (ES-044 AEI, Kimball Physics, USA). The cathode filament was made of tantalum to improve the temperature resistance and extend filament lifetime. To further improve the electron ionization process, a magnet was placed outside the cathodes in order to create a magnetic field. It is a well-known technique to increase electrons' trajectory length, thereby increasing the probability of ionization.

The designed EI ionization source mainly included eight parts: ionization chamber, electron collector, horizontal/perpendicular deflection lens, slit, focus lens group, repeller, cathode filament, magnet, and affiliated insulating PEEK mounting part.

**Figure 3:**
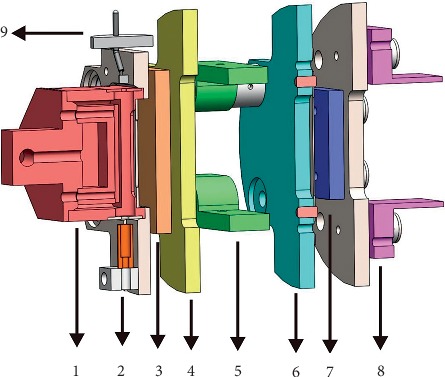
Main composition of the designed EI ionization source. 1: ionization chamber; 2: electron collector; 3: horizontal deflection lens; 4: focus lens; 5: perpendicular deflection lens; 6: slit; 7: horizontal deflection lens; 8: perpendicular deflection lens; 9: cathode filament.

As shown in Figure 3, the ionization chamber was an open structure with an ion ejection hole in the central axis. Specially, the ionization chamber consisted of two hollow semicylindrical structures. A potential difference is formed between the front semicylindrical repeller and the other semicylindrical ionization chamber (Figures 1(e) and 3). The repeller was used to push the ions produced in the ionization chamber by the EI ionization source, which then entered the lens group. The lens group consisted of a horizontal/perpendicular deflection lens, focus lens, and slit.

The electrons generated from the cathode filament entered the ionization chamber through a 2 mm hole, which was favorable for the vacuum increase in the ionization chamber. Compared with the conventional slit-type electron entrance, the grid-type structure had a higher electron transmittance without changing the electric field inside the ionization chamber. Different from the traditional one, our designed EI ionization source adopted an ES-044 AEI source as a cathode filament, which could reduce filament damage of the temperature instability and current fluctuation caused by start and stop filaments instantaneously.

**Figure 4:**
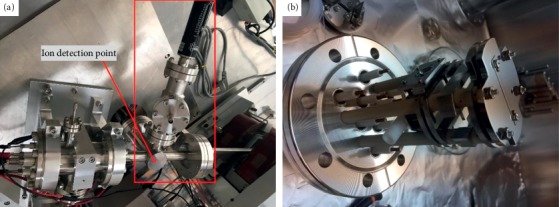
Photography of the homemade EI ionization source apparatus.

Figure 4 shows the photography of a self-developed EI ionization source apparatus which consists of five parts: vacuum system, sample introduction system, EI ionization source, ion transmission system, and ion detector. Gas analytes were directly introduced into the ion source through 100 *μ*m i.d., 1 m long capillaries. The ion source was pumped by a 3.2 L/s dry scroll vacuum pump (Edwards Vacuum Technologies, Inc., UK). The ion optics and the ion detector region were differentially pumped by an 80 L/s and a 300 L/s turbo molecular pump (Pfeiffer Vacuum Technologies, Inc., Germany), respectively. The whole EI ionization source was fixed on a CF63 flange, which is convenient to disassemble and clean the ionization source. The electron beam signal was detected by the weak current-measuring apparatus (6487 Picoammeter, Tektronix, USA).

## 4. Results and Discussion

### 4.1. Electron Intensity and Electron Energy

**Figure 5:**
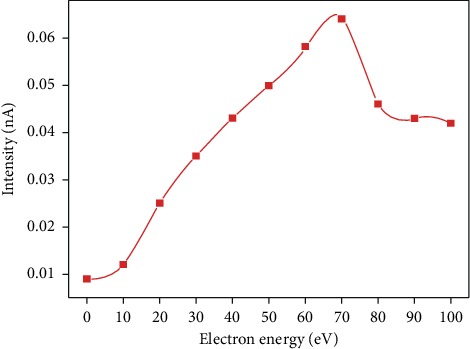
Function of electron intensity with the electron energy emitted from the filament.

The released electron intensity from the cathode filament is an important indicator for evaluating the EI ionization source, which is related to the apparatus performance. Among them, the most direct way to improve the ionization efficiency is to increase the filament current of the EI source; however, the filament lifetime was decreased rapidly. To increase the electron intensity, the influence of the electron energy from the filament on the electron intensity was investigated, as shown in Figure 5.

As the electron energy emitted by the filament increased from 0 to 70 eV, the electron intensity detected by the picoammeter increased slowly at the beginning but accelerated rapidly after 35 eV. When the electron energy emitted by the filament was up to 70 eV, the maximum peak was present. As the electron energy exceeded 70 eV, the detected electron intensity reached a steady state, and then the electron intensity declined continuously. The continuous decline may be attributed to the fact that the high-energy electrons along the vertical axis cannot be received by the detector in a magnetic environment of 150 Gs. The measured curve between electron intensity and electron energy also coincides with that of the electron impact ionization source disclosed by the National Institute of Standards and Technology of the United States.

### 4.2. Electron Intensity and Magnetic Field

**Figure 6:**
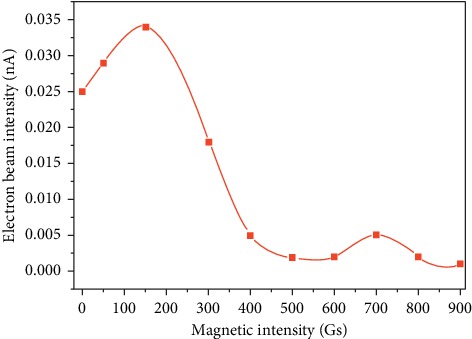
Electron intensity with the various values of the magnetic field from 0 to 900 Gs.

To enhance the probability of ionization by the electron impact of atoms or molecules, a magnet was placed outside the cathode filament to produce a magnetic field inside the ionization chamber. Due to the Lorentz force, the electron trajectory was modified by the magnetic field, thereby increasing the length of the electron trajectory through the ionization chamber. In fact, the spiral radius of the electron trajectory was related to the electron emission energy. To ensure the electron reach the higher intensity, the electron energy emitted by the filament is 70 eV in the following apparatus, which corresponds to the greatest intensity in Figure 5. The results of electron intensity with the various values of the magnetic field from 0 and 900 Gs are shown in Figure 6.

To illustrate the influence of the magnetic field on electron intensity, the investigation was under no magnetic field at the beginning. And then, a magnetic field was applied in the chamber and the electron intensity was increased from 0.025 to 0.03 nA. It was shown that the maximum electron intensity is obtained under the magnetic field of 150 Gs. Electrons are characterized by helical trajectories due to the combined effects of the electrical and magnetic fields. It is well known that the helical trajectories increase the length of their trajectories through the chamber, thereby contributing to a much greater electron density. When magnetic field intensity was further increased, the electron intensity was decreased sharply. It can be attributed to the decrease of the helical trajectory radius, which results in the decrease of scan volume and reduction of the electron density. Moreover, when the intensity of the magnetic field was increased, the helical radius of the electron was decreased, which led to electrons colliding on the chamber wall and failing to reach the detector.

### 4.3. Ion Intensity and Repeller Potential

**Figure 7:**
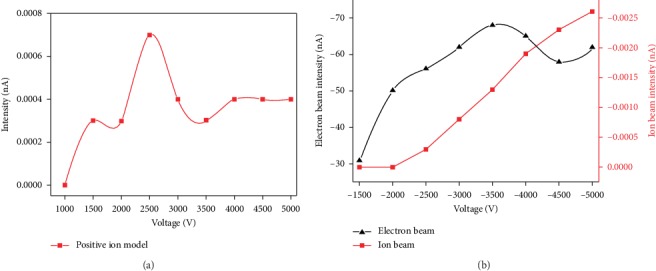
Relationship between ion signal intensity and repeller potential: (a) positive ion mode; (b) negative ion mode.

In addition to the electron parameters, the ion extraction efficiency is another possible parameter influencing the EI ionization source. In this study, the magnetic field is kept constant at 150 Gs and the electron energy emitted from the cathode filament is 70 eV. For the magnetic mass analyzer under development, the potential of the cathode filament was isolated from the electric supply adopted by an isolation transformer. In fact, the potential of the cathode filament was suspended on the repeller potential. In order to extract ions from the ionization chamber, a repeller potential is applied in the direction of ion propagation. The influence of the repeller potential on the molecular ionization was investigated, as shown in Figure 7.

In a positive ion model, the air in the lab was chosen as the test sample, to obtain the optimal parameters; the repeller potential was varied from 1000 to 5000 V, when the repeller potential applied was 2500 V, and the ion intensity attained the maximum value (Figure 7(a)). This fact is consistent with the results described by Ahn and Park [23].

In a negative ion model, the pure oxygen gas was chosen as the test sample; the ion intensity increased when the repeller potential varied from −1000 to −5000 V. During lower repeller potential, the air obstruction decreases the amount of ion beam flying off from the ionization chamber. When the repeller ability is above −2000 V, the ion intensity flew off from the ionization chamber gradually (Figure 7(b)). But enslaved to the mass analysis ability of the mass analyzer, the repeller potential was not the greater the better.

## 5. Conclusion

This study was focused on numerical simulations and design of a homemade electron impact ion source, which was equipped with a magnet and new structure to enhance the electron intensity and ionization efficiency. Based on the ion optic mechanism, we have designed the electron impact ion source. Numerical simulation and geometry were optimized by SIMION 8.0 to provide higher electron intensity and ion transmission efficiency. To improve the electron intensity, the influence of the cathode filament potential and magnetic intensity was investigated, and the values of 70 eV and 150 Gs were chosen in our apparatus. The maximum electron intensity with the filament potential at 70 eV can be attributed to the fact that the high-energy electrons along the vertical axis cannot be received by the detector. The maximum electron intensity with the magnetic intensity at 150 Gs can be attributed to the decrease of the helical trajectory radius, which results in the decrease of scan volume and reduction of the electron density. Based on optimal parameters, the air in the lab and pure oxygen gas were detected by the homemade apparatus, and the ion intensity was detected in the positive and negative ion modes, respectively. This design has the potential to enhance ionization efficiency applied in the mass spectrometer ionization source.

## Data Availability

The data used to support the findings of this study are available from the corresponding author upon request.

## Supplementary Materials

Supplementary Materials Figure S1: (a) electron beam distribution, initial energy distribution, and emission direction set in ion optic software SIMION 8.0. A simulated electron emission condition was close to the real circumstance; (b) ion beam distribution, initial energy distribution, and emission direction set in ion optic software SIMION 8.0. A simulated ion emission condition was close to the real circumstance; (c) initial potential of the repeller, deflection lens, and focus lens in ion optic software SIMION 8.0.
