# Supplementary material for: Numerical Simulations and the Design of Magnetic Field-Enhanced Electron Impact Ion Source with Hollow Cylinder Structure
Source: J Anal Methods Chem. 2020 Jan 25;2020:2809485. doi: 10.1155/2020/2809485 (PMC7204179; doi:10.1155/2020/2809485)

FIGURE S1：(a)Electron beam simulation parameter using in SIMION 8.0;


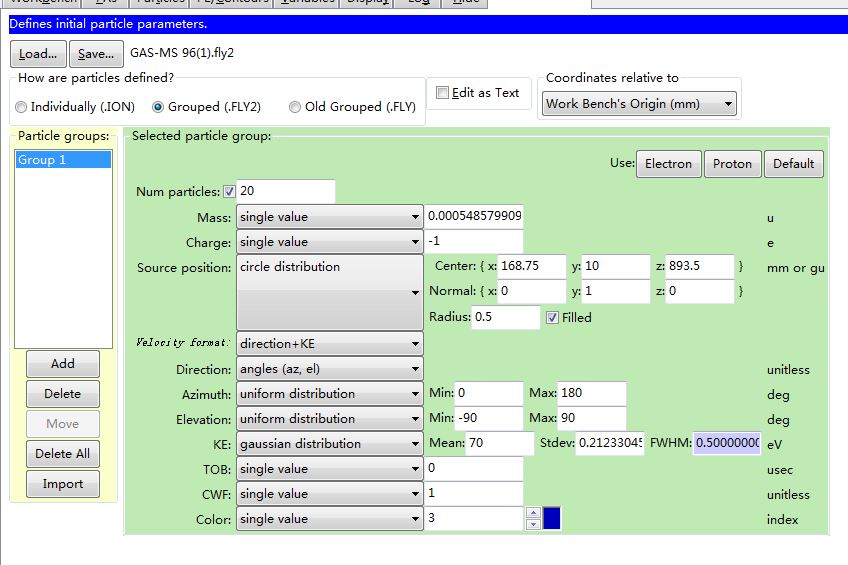


（b）Ion beam simulation parameter using in SIMION 8.0；


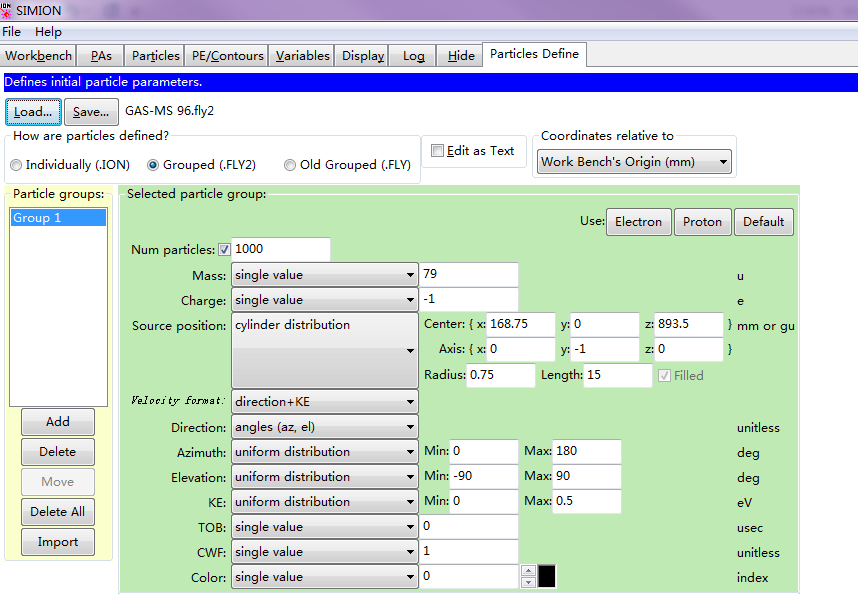


(c) Simulation parameter of EI ionization source using in SIMION 8.0.


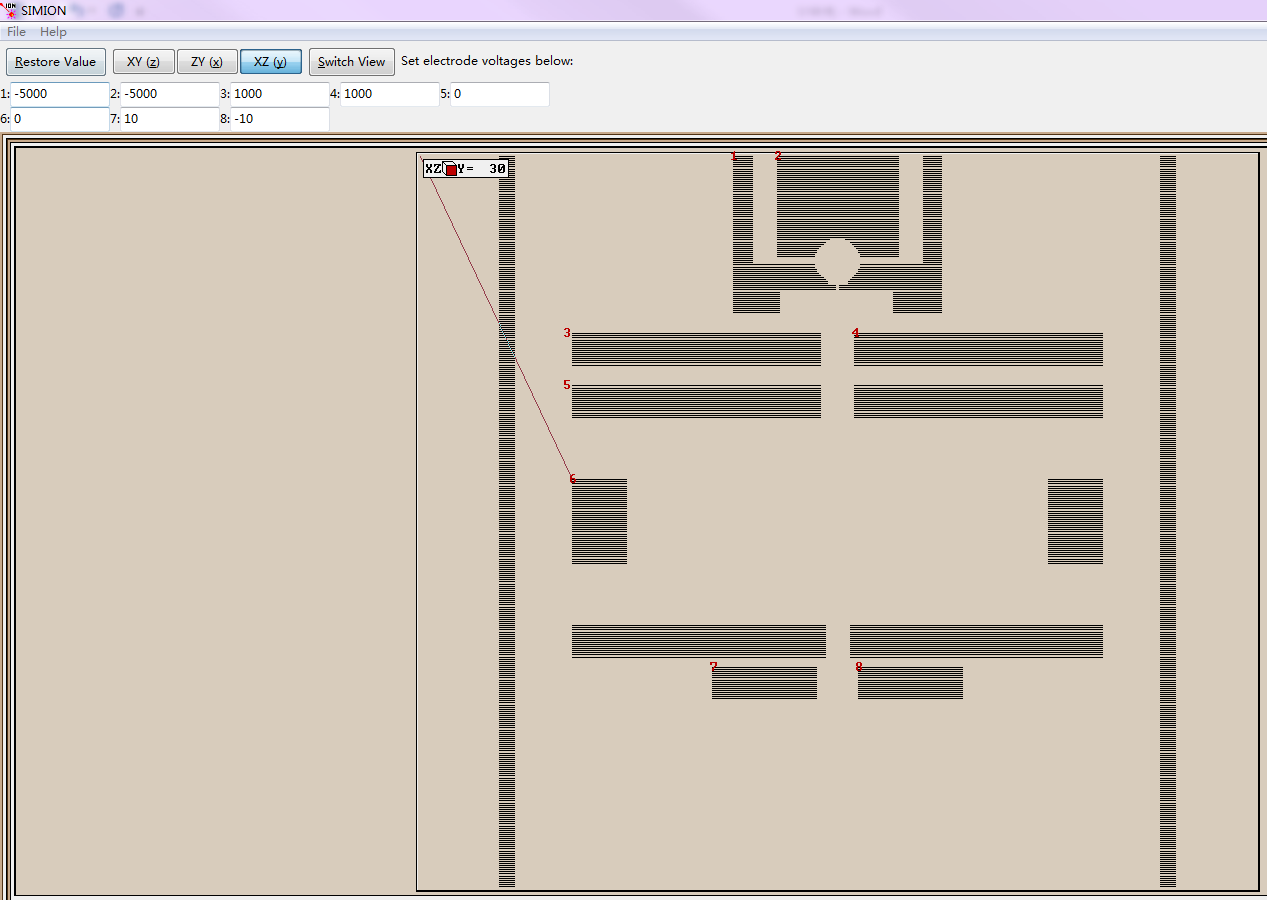

Supplement: Supplementary Materials — Figure S1: (a) electron beam distribution, initial energy distribution, and emission direction set in ion optic software SIMION 8.0. A simulated electron emission condition was close to the real circumstance; (b) ion beam distribution, initial energy distribution, and emission direction set in ion optic software SIMION 8.0. A simulated ion emission condition was close to the real circumstance; (c) initial potential of the repeller, deflection lens, and focus lens in ion optic software SIMION 8.0. [file 2809485.f1.docx]
